# First record of *Odontosphaeropyx* Cameron, 1910 from the Oriental Region with description of a new species from Thailand (Hymenoptera, Braconidae, Cheloninae)

**DOI:** 10.3897/zookeys.809.30742

**Published:** 2018-12-19

**Authors:** Donald L.J. Quicke, Buntika A. Butcher

**Affiliations:** 1 Integrative Ecology Laboratory, Department of Biology, Faculty of Science, Chulalongkorn University, Phayathai Road, Pathumwan, BKK 10330, Thailand Chulalongkorn University Pathumwan Thailand; 2 Center of Excellence in Entomology: Bee Biology, Diversity of Insects and Mites, Chulalongkorn University, Phayathai Road, Pathumwan, BKK 10330, Thailand Chulalongkorn University Pathumwan Thailand

**Keywords:** Cheloninae, parasitoid, Afrotropical, Oriental, range extension, new taxon

## Abstract

*Odontosphaeropyxmatasi* Quicke & Butcher, **sp. n.** from Thailand is described and illustrated. The new species represents the first known record of *Odontosphaeropyx* from outside of the Afrotropical Region. A key is provided to separate it from the apparently closely related *O.flavifasciatus* Zettel, 1990, with which it shares almost identical colouration, very different from the other five known species.

## Introduction

The Cheloninae is a large cosmopolitan subfamily of ovo-larval parasitoids of Lepidoptera (Shaw and Huddleston 1991, Quicke 2015). It is dominated in terms of numbers of species by three genera, *Ascogaster* Wesmael, 1835, *Chelonus* Panzer, 1806, and *Phanerotoma* Wesmael, 1838, which are collectively represented by more than 1500 described species (Yu et al. 2016). However, worldwide another 16 genera (including the Adeliini) are known, most with more restricted geographic distributions and often known from only a few species and specimens (Zettel 1990, He et al. 1994, Kittel and Austin 2014). The OdontosphaeropyginiZettel 1990 (= PseudophanerotominiZettel 1990, synonymized by Braet et al. 2014) are a monotypic tribe with six included species, all in *Odontosphaeropyx* Cameron, and all from the Afrotropical region (Kittel et al. 2016). Here we report the discovery of the first species from outside of Africa and describe a new species of *Odontosphaeropyx* of Thailand.

The discovery of the new species was the result of the extensive TIGER (Thailand Inventory Group for Entomological Research) programme which sampled insects in 25 national parks in Thailand over a three-year period, 2006–2008 (see http://sharkeylab.org/tiger). This programme yielded many thousands of braconid wasp specimens. Among these we discovered two specimens of a relatively large-bodied chelonine, with a single clypeal tooth, fore wing vein (RS+M)a arising from vein 1-M well removed from parastigma, forewing vein m-cu joining (RS+M)a basal to 2RS, and with a pair of transverse sutures on the carapace. Initial generic identification based on the known Asian fauna was problematic, so we used the key to world genera and tribes of Zettel (1990) and obtained a clear identification as *Odontosphaeropyx* which was previously known from six described species, all from sub-Saharan Africa and Madagascar (Braet et al. 2014).

## Materials and methods

Terminology follows van Achterberg (1988) except for wing venation nomenclature which follows Sharkey and Wharton (1997); see also fig. 2.2 in Quicke (2015) for comparison of wing venation naming systems.

Specimens were imaged using an Olympus SXZ16 microscope with automated multiple image capture at pre-set focal levels using an Olympus DP72 camera, and image combination using the Cell^D image processing system.

Collection abbreviations: **CUMZ** (Collection of the Insect Museum, Chulalongkorn University Museum of Natural History, Bangkok); **QSBG** (Queen Sirikit Botanic Gardens, Chiang Mai, Thailand.

## Species description

### Odontosphaeropyx matasi

Taxon classificationAnimaliaHymenopteraBraconidae

Quicke & Butcher sp. n.

http://zoobank.org/23D59C0D-8E85-4921-91CA-22A2D48E0F7A

Fig. 1

#### Type material.

**Holotype** male, THAILAND: Kamphaeng Phet Mae Wang NP, 3–10.ix.2007, 1306 m, C Puluk, A Inpuang, T2812 (QSBG). **Paratype** male, same data as holotype (CUMZ).

#### Diagnosis.

The new species can be distinguished from all other *Odontosphaeropyx* species in having the combination of an orange thorax, a largely black metasoma with white-banded 2^nd^ tergite, and fore wing vein 3RSa longer than r-rs.

#### Description.

Length of body 7.2 mm, of fore wing 6.0 mm and of antenna 6.2 mm. Antenna with 38 flagellomeres. Penultimate flagellomere 1.8× longer than wide. First flagellomere 1.3× longer than 2^nd^; 3.3× longer than wide. Scapus with ‘v’-shaped notch on outer apical margin. Antennal sockets distinctly above level of top of eye. Width of head 1.3× length of head in lateral view. Eyes 2.0× taller than wide in frontal view; glabrous. Width of head: height of eye: width of face = 2.6: 1.0: 1.6. Face and clypeus with dense setiferous punctation. Intertentorial distance 2.0× tentorio-ocular distance. Clypeus produced into a strong median tooth. Length of temple 1.3× length of eye in dorsal view. Frons demarked by a sharply-defined elevation running from front of eye straight to and around stemmaticum; with a crescent-shaped ridge in front of anterior ocellus. Occipital carina complete.

Notauli deeply impressed, foveate-crenulate, the area between them on posterior half of mesoscutum depressed (lower than lateral lobes) and evenly strongly rugose. Scutellar sulcus curved, deep and with 4–6 strong crenulae between outer pair. Mesopleuron and mesosternum with small, dense, setiferous punctures, the cuticle between the punctures shiny. Median area of metanotum with complete mid-longitudinal carina. Propodeum with distinct apophyses, a wide medial groove bordered by irregular carinae and transversed by a ladder-like set of carinae superimposed on rugose background.

Fore wing. Vein 1CUb 3.1× longer than 1CUa. Lengths of veins r-rs: 3RSa: 3RSb = 1.0: 1.3: 4.9.

Length of fore femur: tibia: tarsus = 1.0: 1.50: 1.30. Length of hind femur: tibia: tarsus = 1.0: 1.25: 1.15. Hind femur 4.4× longer than maximally deep. Claws with a pectin of two teeth.

Metasoma 2.8× longer than maximally wide. First tergite with strong, though somewhat irregular, dorsal carinae that almost meet the posterior margin of the tergite. Sutures between the three carapace segments well developed.

Coloration. Head, palps, propleuron, ventromedial part of mesosternum, metapleuron (mostly), propodeum, metasoma except most of second tergite, legs except fore tibia black; fore tibia cream-coloured; 2^nd^ metasomal tergite except medio-posteriorly, white. Wings hyaline with a pale brown cross-band at level of parastigma and pale brown distally from slightly beyond base of pterostigma.

Female. Not known.

**Figure 1.**
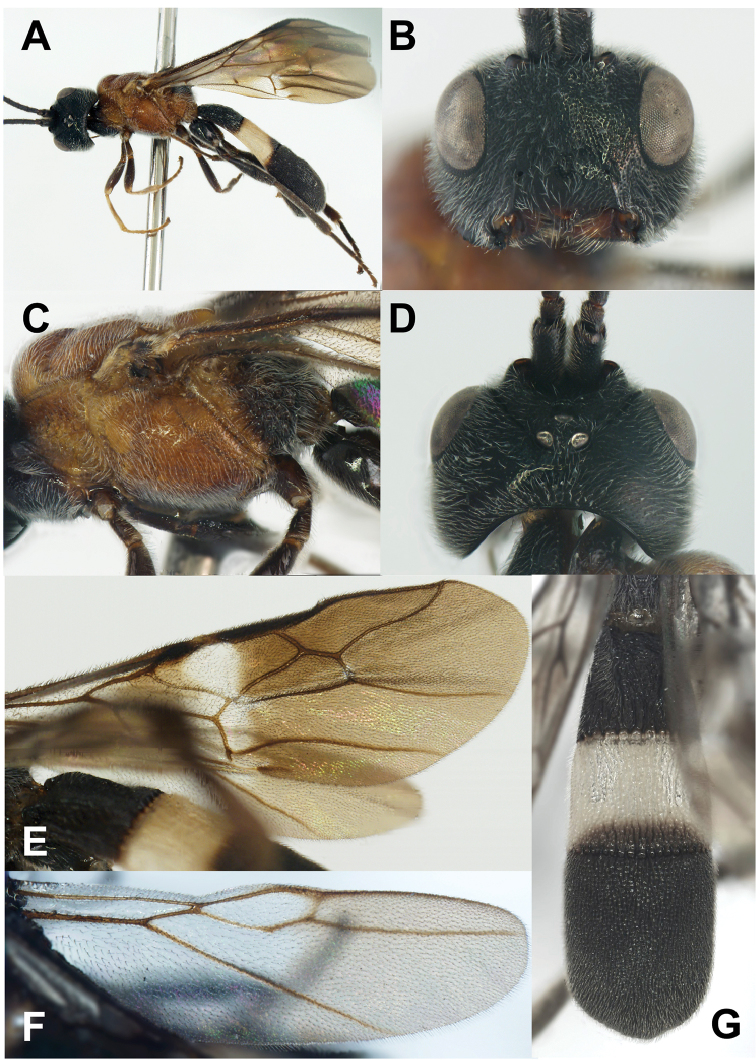
*Odontosphaeropyxmatasi* sp. n., males. **A** holotype, habitus lateral view **B** holotype, head, front view **C** paratype, mesosoma, lateral view **D** holotype, head, dorsal view **E** paratype, fore wing **F** holotype, hind wing **G** paratype, metasoma, dorsal view.

#### Biology.

Not known.

#### Variation.

Paratype. Vein 1CUb 3.3× longer than 1CUa. Lengths of veins r-rs: 3RSa: 3RSb = 1.0: 1.25: 4.8. Otherwise almost identical to holotype.

#### Etymology.

Named after Mr Matas Srisabye, late friend, triathlete, Thai National Team athlete (water polo) and running coach of BAB.

#### Remarks.

In the key to *Odontosphaeropyx* species by Braet et al. (2014), which was modified after the one by Zettel (1990), this new species falters at couplet 1 because it has fore wing vein 3RSa longer than r-rs but has the metasoma more than 2.6× longer than wide. The only described species with similar colouration (orange thorax and largely black metasoma with white-banded 2^nd^ tergite) is *O.flavifasciatus* Zettel, 1990, which is known from Nigeria (type locality) and Democratic Republic of Congo. The two species may be separated using the following amended couplet:

**Table:** 

1	Fore wing vein 3RSa longer than r-rs; mesopleuron punctate; fore wing vein 1CUb approximately 3× longer than 1CUa; fore tibia cream-coloured	***O.matasi* sp. n.**
–	Fore wing vein 3RSa shorter than r-rs; mesopleuron strongly foveate-rugose; fore wing vein 1CUb approximately 2× longer than 1CUa; fore tibia black	*** O. flavifasciatus ***

In addition to the description and drawings of the holotype of *O.flavifasciatus* [as *Pachychelonusflavofasciatus* Zettel] given in Zettel (1990: figs 3–9), Braet et al. (2012: figs 48–51) provide photographs of a specimen from Democratic Republic of Congo, and further images of the holotype are on the WaspWeb web site housed at the Iziko institution (http://www.waspweb.org/) (accessed 30 September 2018).

## Discussion

The increasing use of Malaise traps in diverse countries is resulting in major range extensions of many braconid taxa (e.g., Sharkey 2004, Tan et al. 2010, Sharkey and Braet 2012, Kittel and Austin 2013, Butcher et al. 2016, Ranjith et al. 2017). Given this, together with the relative paucity of studies on SE Asian Braconidae, it is not too surprising that a principally Afrotropical genus also occurs there. Until 2016 only 373 Braconidae species had been recorded from Thailand (Yu et al. 2016) of which 199 belong to the Rogadinae (largely by BA Butcher and collaborators) and 70 to the Agathidinae (largely as a result of Mike Sharkey’s studies). Material for both of these groups mainly originated from the TIGER (Thailand Inventory Group for Entomological Research) project. Since much of the TIGER material has yet to be systematically investigated taxonomically, with most braconid subfamilies hardly investigated, it is likely that the Thai braconid fauna will eventually be found to be several times larger than the current total.

## Supplementary Material

XML Treatment for Odontosphaeropyx matasi
